# Quantitatively Evaluating the Impact of Eliminating Risk Assessment Checklists for Granting Leave in a Specialist Personality Disorder Ward

**DOI:** 10.1192/bjo.2022.413

**Published:** 2022-06-20

**Authors:** Alasdair Philbey

**Affiliations:** Cambridge University, Cambridge, United Kingdom

## Abstract

**Aims:**

Springbank Ward, in the CPFT NHS trust, is a specialist unit for patients with a diagnosis of emotionally unstable personality disorder (EUPD). Psychiatric wards often use restrictive practices to try and minimise suicide risk. Using risk assessment checklists to decide whether to grant leave is one example. Research shows that it is not possible to predict suicide or self-harm risk at an individual level, regardless of the assessment method used, so we questioned the utility of such an approach. A previous evaluation of our leave protocol showed that patients and staff would favour a less restrictive and more personalised approach. We introduced a new protocol that eliminated use of checklists, replacing them with an optional 1:1 conversation with staff before leaving the ward. Our aim in this service evaluation was to determine whether there was any significant change in rates of incidents on the ward and during leave as a result of this new, less restrictive leave protocol.

**Methods:**

Data were obtained from the records of incidents on Springbank ward from March 2019 to March 2021. These incidents were recorded by members of staff on the ward, and ranked according to the severity of harm that resulted from these incidents. The rankings from least severe to most severe recorded during the study were ‘No harm’, ‘Low (Minimal Harm)’, and ‘Moderate (Short term harm)’. The number of incidents which occurred for the year before and the year after the policy change were compared. The comparison compared both the total amount of incidents and the sub-types of incidents.

**Results:**

In the 365 days following the change in protocol, there was a 15.5% decrease in total incidents and a 51.0% decrease in incidents occurring off the ward compared to the 365 days before the change in protocol. Notably there was a 61% decrease in total (both on and off the ward) Moderate (Short term harm) incidents, the most harmful type of incident recorded, following the change in protocol.

**Conclusion:**

The decrease in incidents following the change in protocol suggests that replacing the use of a formal risk assessment checklist with a holistic alternative improves patient safety.

